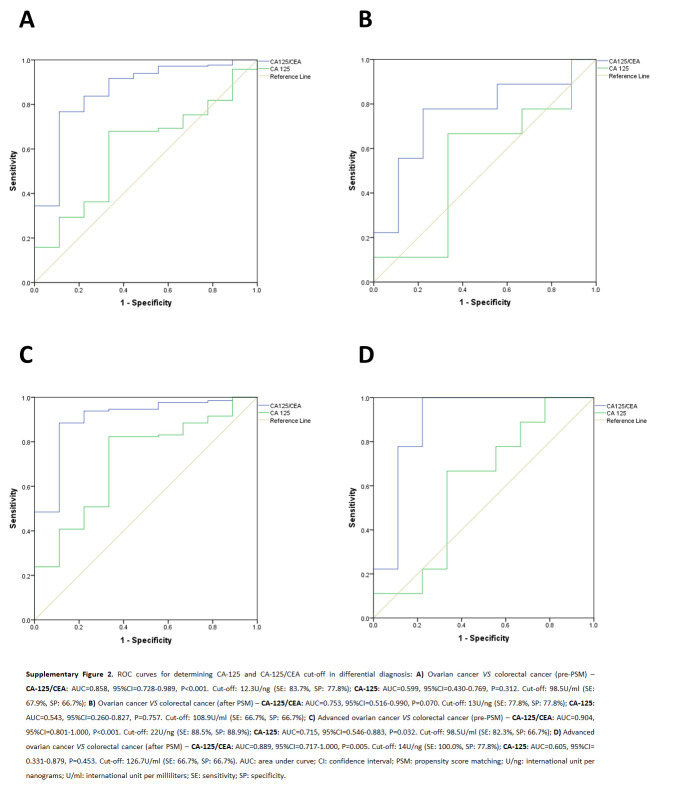# Can CA-125/CEA ratio be used for the differential diagnosis between ovarian and nonovarian cancers? A research letter

**DOI:** 10.1097/JS9.0000000000002015

**Published:** 2024-08-14

**Authors:** Jeferson R. Zanon, Elisa B. Simioni, Flávia F. Barbin, Priscila G. Pedrão, Carlos E.M.C. Andrade, Diocésio A.P. de Andrade, João P. Bilibio, José H.T.G. Fregnani, Ricardo dos Reis

**Affiliations:** a Department of Nephrology and Palliative Care at Jales Cancer Hospital (a Barretos Cancer Hospital Unit – Pio XII Foundation), Jales; b Department of Gynecologic Oncology at Jaú Cancer Hospital (Amaral Carvalho Foundation), Jaú; c Department of Pathology at Barretos Cancer Hospital (Pio XII Foundation); d Molecular Oncology Research Center, Barretos Cancer Hospital (Pio XII Foundation); e Department of Gynecologic Oncology at Barretos Cancer Hospital (Pio XII Foundation), Barretos; f Clinical Oncology Department, InORP Oncoclínicas Group, Ribeirão Preto, São Paulo; g Brusque Medical School (UNIFEBE), Brusque, Santa Catarina; h A.C. Camargo Cancer Center, São Paulo; i Graduate Program at Barretos Cancer Hospital (Pio XII Foundation), Barretos, São Paulo; j Department of Gynecologic Oncology; k Educational and Research Institute; l Graduate Program at Barretos Cancer Hospital (Pio XII Foundation), Barretos, São Paulo, Brazil

Highlights

- CA-125/CEA is more accurate than CA-125 for the differential diagnosis of ovarian cancer.
- Propensity score matching promoted better results in all comparisons.
- CA-125/CEA may only be useful to differentiate advanced ovarian from advanced colorectal cancer.

It is known that ovarian cancer ranks fourth in incidence, third in mortality, and first in lethality among gynecological neoplasms worldwide^1^. There is a lack of accurate methods for early detection of this tumor, and the most used serum tumor marker, CA-125, has limited sensitivity and specificity for early diagnosis^2^. Ovaries can also be affected by other metastatic tumors, hampering the diagnosis and delaying the beginning of treatment^3^. Since 1988, studies have evaluated the role of the CA-125/CEA ratio in the differential diagnosis of women with malignant pelvic masses^4–7^. They have shown that the CA-125/CEA is a simple, noninvasive method that can aid in the preoperative evaluation, particularly in differentiating women with nonovarian cancers. However, the cutoffs were not the same^4–7^. Our research aimed to assess whether the CA-125/CEA ratio is a diagnostic tool for the differential diagnosis between ovarian cancer and other advanced intra-abdominal neoplasms.

After approval by the local institutional review board, a cross-sectional study design was conducted of patients treated at the gynecologic oncology department of a specialized tertiary hospital with initial suspicion of ovarian malignancy between May 2001 and July 2018. Patients with pelvic mass, peritoneal carcinomatosis, and/or ascites were included, and serum CA-125 and CEA were obtained at the patient’s first clinical visit. Patients were excluded if the pathology results showed benign lesions. Five groups were formed: 1 – patients with ovarian cancer (clinical stages I–IV); 2 – patients with advanced clinical stage (III or IV) ovarian cancer; 3 – patients with advanced (stage IV) colorectal cancer; 4 – patients with gastrointestinal cancer (gastric and colorectal cancers); 5 – patients with other nonovarian cancer (gastric, colorectal, and other cancers). These groups were compared two by two with the ovarian cancer group (advanced or not). Nonparametric variables are described as median and respective interquartile range, the Mann–Whitney test was used to compare two independent samples. Categorical variables were presented in absolute and relative frequencies using *χ* ^2^ tests of association or Fisher’s exact tests. Propensity score matching (PSM) analysis was performed after the initial association analysis between groups, calculated by a logistic regression model for each of the five groups mentioned above. Receiver operating characteristic (ROC) curves were performed to determine the cut-off values for both CA-125 and CA-125/CEA ratios in the participants. A subsequent performance characteristic test was performed using pathology findings as the gold standard. All analyses were performed using IBM SPSS v.26.0 software at a 95% confidence level.

There were 262 malignant cases, 47 (17.9%) of nonovarian origin, of which 9/47 (19.2%) were colorectal cancers (Fig. 1). The proportion of white patients was higher in the ovarian cancer group than in all other groups, with statistical significance. There was no difference in terms of BMI, presence of ascites, peritoneal carcinomatosis, pelvic mass, and hormonal status of these patients in all groups (Table 1). The median CA-125/CEA was higher in the ovarian group (145.8 U/ng) than in the nonovarian (22.7 U/ng; *P*<0.001), gastrointestinal (22.7 U/ng; *P*<0.001), and advanced colorectal (2.6 U/ng; *P*<0.001) groups. CA-125/CEA was also higher in the advanced ovarian group than in the advanced colorectal group, 374.4 U/ng versus 2.6 U/ng, respectively (*P*<0.001), as shown in Table 1. The best sensitivity (88.5%) and specificity (88.9%) for pre-PSM analysis was obtained for advanced ovarian cancer (stage III or IV) compared to advanced colorectal cancer within CA-125/CEA higher than 22 U/ng. The positive likelihood ratio was 7.97 and the negative likelihood ratio was 0.13. After PSM analysis, the sensitivity for advanced ovarian cancer with a CA-125/CEA value higher than 14 U/ng was 100% compared to advanced colorectal cancer. Comparisons with the other groups showed low sensitivity and specificity (Table 2).

**Fig. 1:**
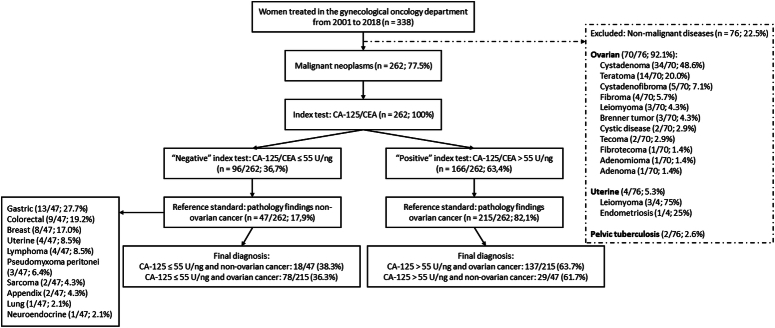
Patients with pelvic mass, peritoneal carcinomatosis, and/or ascites referred to the department of gynecologic oncology.

**Table 1:**
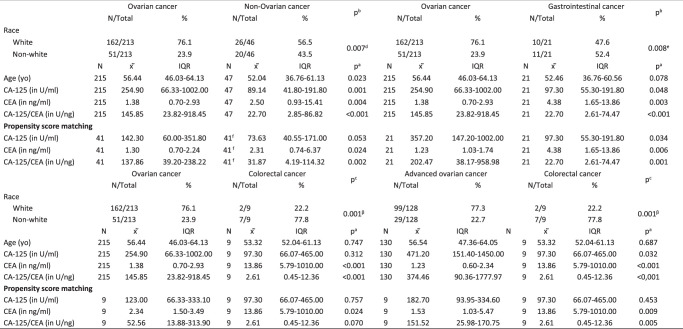
Clinical and laboratory characteristics patients presenting with malignant pelvic, ascites, and/or peritoneal carcinomatosis at diagnosis.

*N*, absolute frequency; %, relative frequency; *x ̃*, median; IQR, interquartile range.

^a^ Mann–Whitney test for continuous variable.

^b^ *χ* ^2^ test.

^c^ Fisher’s exact test for categorical variables.

^d^ Three missing data.

^e^ Two missing data.

CA-125, carbohydrate antigen 125; CEA, carcinoembryonic antigen; CA-125/CEA, CA-125 and CEA ratio; ml, milliliter; ng, nanogram; U, unit; yo, years old.

^f^ Nonovarian group: 12/41 (29.3%) – Gastric cancer; 9/41 (22.0%) – Colorectal cancer; 5/41 (12.2%) – Breast cancer; 4/41 (9.8%) – Uterine cancer; 3/41 (7.3%): Lymphoma; 3/41 (7.3%) – Pseudomyxoma peritonei; 2/41 (4.9%) – Appendix cancer; 2/41 (4.9%) – Sarcoma; 1/41 (2.4%) – Lung cancer.

**Table 2:**
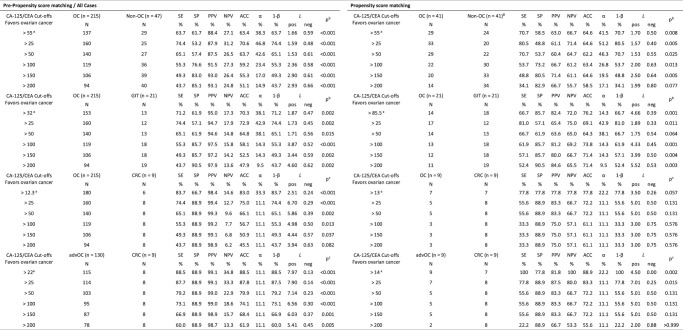
Tests performance characteristics of ovarian cancer for different CA-125/CEA ratio cutoffs.

CA-125, carbohydrate antigen 125; CEA, carcinoembryonic antigen; CA-125/CEA, CA-125 and CEA ratio (in U/ng); *N*, absolute frequency; %, relative frequency; OC, ovarian cancer; GIT, gastrointestinal tract cancer; advOC, advanced ovarian cancer (III or IV clinical stage); CRC, colorectal cancer

θ: Nonovarian group, 12/41 (29.3%) – Gastric cancer; 9/41 (22.0%) – Colorectal cancer; 5/41 (12.2%) – Breast cancer; 4/41 (9.8%) – Uterine cancer; 3/41 (7.3%) – Lymphoma; 3/41 (7.3%) – Pseudomyxoma peritonei; 2/41 (4.9%) – Appendix cancer; 2/41 (4.9%) – Sarcoma; 1/41 (2.4%) – Lung cancer.

α, type 1 error; 1-β, 1 minus type 2 error; ACC, accuracy; L, positive and negative likelihood ratio; NPV, negative predictive value; PPV, positive predictive value; SE, sensitivity; SP, specificity.

^a^ Value determined using ROC curve (see Supplementary Material, Supplemental Digital Content 1, http://links.lww.com/JS9/D303, SDC Fig. S1, Supplemental Digital Content 2, http://links.lww.com/JS9/D304 and SDC Fig. S2, Supplemental Digital Content 3, http://links.lww.com/JS9/D305); <: less than; >: greater than.

^b^ *χ* ^2^ test.

^c^ Fisher’s exact test.

We tested six different thresholds for the differential diagnosis of advanced or nonadvanced ovarian cancer and other neoplasms grouped into nonovarian, gastrointestinal, and advanced colorectal cancers. An increase in specificity was observed when narrowing the comparisons only to those between ovarian cancer and colorectal cancer. The worst results were obtained when comparing ovarian and nonovarian cancers. Studies comparing the CA-125/CEA ratio between ovarian and nonovarian cancers have found different cutoffs with varying sensitivities and specificities: CA-125/CEA less than 10 with 100% specificity^4^; CA-125/CEA greater than 25 with 100% specificity^5^; CA-125/CEA greater than 25 with 62.6% specificity^6^, and CA-125/CEA greater than 100 with 84.1% specificity^6^. Yedema *et al*.^7^ analyzed patients with ovarian cancer and women with colorectal cancer. They found that for a CA-125/CEA ratio greater than 25, there was a sensitivity of 91% and a specificity of 100%. The heterogeneity of the populations and the different sample sizes for metastatic disease to the ovary in these studies, including ours, may explain all these differences in the literature results. One clinical trial^8^ used the CA-125/CEA ratio with an imaging test as one of the eligibility criteria for a population with advanced ovarian cancer. This association between the CA-125/CEA ratio and imaging studies has not been previously studied. Nunes Pereira *et al*.^9^ found that in women with an ultrasound scan showing an adnexal mass of ovarian origin, a resonance scan combined with a CA-125/CEA ratio greater than 25 had a 100% sensitivity. Our research is limited by the observational nature of the design, and a prospective analysis could clarify our findings. Unfortunately, we had only nine patients with colorectal cancer, which may also affect the results. However, in light of the ESMO-ESGO 2019 consensus^10^ recommending complete tumor resection at initial debulking as the most important prognostic factor in patients with advanced ovarian cancer, this tool may be useful in guiding surgical decisions. The CA-125/CEA ratio is certainly not a decisive factor in the diagnosis of ovarian cancer, but it has great potential for ruling out other types of cancer, especially colorectal cancer. We believe that a CA-125/CEA ratio may help to differentiate advanced ovarian cancer (stage III or IV) from stage IV colorectal cancer, but it is not a reliable tool for differentiating ovarian cancer from other types of cancer in women with advanced intra-abdominal neoplasms, presenting with a pelvic mass and/or peritoneal carcinomatosis and/or ascites.

## Ethical approval

This study was approved by the local institutional review board, Barretos Cancer Hospital Research Ethics Committee under the number 54129515.8.0000.5437.

## Consent

This study is not a case report. Nonetheless a waiver of informed consent was granted by the local institutional review board.

## Source of funding

There were not any financial support and sponsorship.

## Author contribution

J.R.Z.: formal analysis, data curation, writing – original draft, and writing – review and editing; E.B.S.: methodology, investigation, data curation, project administration, and writing – review and editing; F.F.B.: validation, writing – review, and editing; P.G.P., C.E.M.C.A., D.A.P.d.A., and J.P.B.: writing – review and editing; J.H.T.G.F.: formal analysis, writing – review, and editing; R.d.R.: conceptualization, methodology, writing – original draft, writing – review and editing, and supervision.

## Conflicts of interest disclosure

The authors declare that they have no financial conflicts of interest with regard to the content of this report.

## Research registration unique identifying number (UIN)

1. Name of the registry: ISRCTN registry platform (https://www.isrctn.com).
2. Unique identifying number or registration ID: ISRCTN15248104.
3. Hyperlink to your specific registration (must be publicly accessible and will be checked): https://doi.org/10.1186/ISRCTN15248104

## Guarantor

Jeferson Rodrigo Zanon and Ricardo dos Reis.

## Data availability statement

The data that support the findings of this study are available from the corresponding author, J.R.Z., upon reasonable request.

## Provenance and peer review

There was no invitation from any journal for this article.

## Supplementary Material

**Figure:**
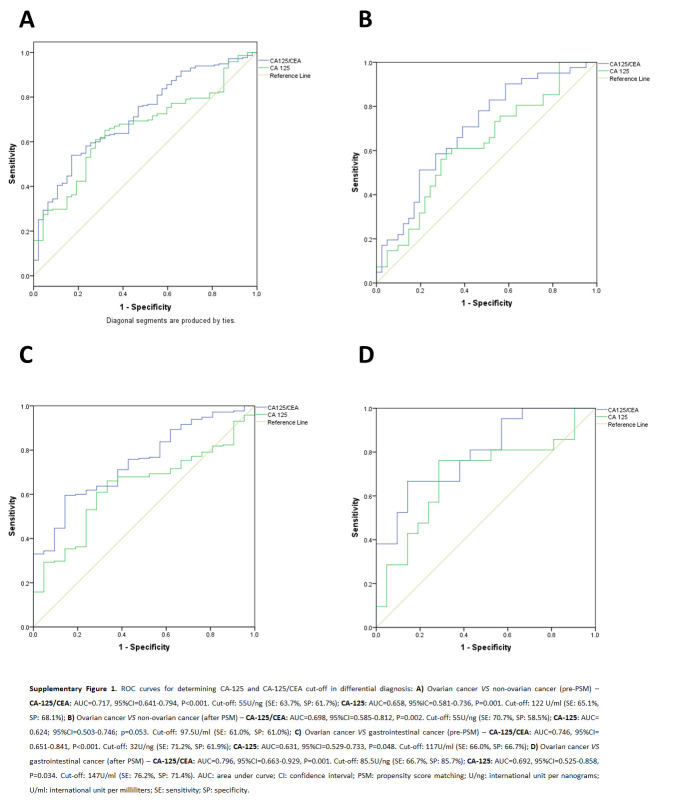


**Figure:**