# Supplementary material for: Can CA-125/CEA ratio be used for the differential diagnosis between ovarian and nonovarian cancers? A research letter
Source: Int J Surg. 2024 Aug 14;110(11):7397–400. doi: 10.1097/JS9.0000000000002015 (PMC11573086; doi:10.1097/JS9.0000000000002015)
Supplement: Supplementary file 1 [file js9-110-7397-s001.docx]

**Can CA-125/CEA ratio be used for the differential diagnosis between ovarian and non-ovarian cancers? A research letter**

**Supplementary material**

*General analysis comparing CA125/CEA versus CA125 for differential diagnosis*

The pre-PSM univariate ROC analyses showed that the area under the curve (AUC) of the CA-125/CEA ratio was higher than the AUC of CA-125 for the diagnosis of ovarian cancer compared to the other groups. The AUC was higher for CA-125/CEA when comparing advanced ovarian cancer with advanced colorectal cancer (Supplementary Figure 1A, Supplementary Figure 1C, Supplementary Figure 2A, and Supplementary Figure 2C). After PSM, there was no significant difference only in the comparison between ovarian cancer and colorectal cancer, although the AUC of CA-125/CEA was higher than the AUC of CA-125 (Supplementary Figure 2B).

We found that the CA-125/CEA ratio was superior to CA-125 alone for the diagnosis of ovarian cancer. CA-125 alone is not used for the diagnosis of ovarian cancer, especially for early diagnosis, because in the early stages CA-125 is elevated in half of the cases^1^. A meta-analysis showed that the sensitivity of using CA-125 (cut-off value of 35 U/ml) for the diagnosis of ovarian cancer was 79% (95% CI: 77-82%) and the specificity was 78% (76-80%)^2^. Our results were even lower for a CA-125 cut-off value of 122 U/ml to differentiate ovarian from non-ovarian cancer. The main recommendation of CA-125 is for the assessment of response and progression of ovarian cancer in combination with clinical and radiological assessment^3^.

In this study, CA-125/CEA ratio was more accurate than CA-125 in differentiating ovarian cancer from other pelvic malignancies.

**REFERENCES**

1. Hellstrom I, Raycraft J, Hayden-Ledbetter M, et al. The HE4 (WFDC2) protein is a biomarker for ovarian carcinoma. 2003;63(13):3695-3700.

2. Ferraro S, Braga F, Lanzoni M, Boracchi P, Biganzoli EM, Panteghini MJJocp. Serum human epididymis protein 4 vs carbohydrate antigen 125 for ovarian cancer diagnosis: a systematic review. 2013;66(4):273-281.

3. Colombo N, Sessa C, du Bois A, et al. ESMO–ESGO consensus conference recommendations on ovarian cancer: pathology and molecular biology, early and advanced stages, borderline tumours and recurrent disease. 2019;30(5):672-705.

**FIGURE LEGENDS**

**Supplementary Figure 1.** ROC curves for determining CA-125 and CA-125/CEA cut-off in differential diagnosis: **A)** Ovarian cancer *VS* non-ovarian cancer (pre-PSM) – **CA-125/CEA:** AUC=0.717, 95%CI=0.641-0.794, P<0.001. Cut-off: 55U/ng (SE: 63.7%, SP: 61.7%); **CA-125:** AUC=0.658, 95%IC=0.581-0.736, P=0.001. Cut-off: 122 U/ml (SE: 65.1%, SP: 68.1%); **B)** Ovarian cancer *VS* non-ovarian cancer (after PSM) – **CA-125/CEA:** AUC=0.698, 95%CI=0.585-0.812, P=0.002. Cut-off: 55U/ng (SE: 70.7%, SP: 58.5%); **CA-125:** AUC=0.624; 95%CI=0.503-0.746; p=0.053. Cut-off: 97.5U/ml (SE: 61.0%, SP: 61.0%); **C)** Ovarian cancer *VS* gastrointestinal cancer (pre-PSM) – **CA-125/CEA:** AUC=0.746, 95%CI=0.651-0.841, P<0.001. Cut-off: 32U/ng (SE: 71.2%, SP: 61.9%); **CA-125:** AUC=0.631, 95%CI=0.529-0.733, P=0.048. Cut-off: 117U/ml (SE: 66.0%, SP: 66.7%); **D)** Ovarian cancer *VS* gastrointestinal cancer (after PSM) – **CA-125/CEA:** AUC=0.796, 95%CI=0.663-0.929, P=0.001. Cut-off: 85.5U/ng (SE: 66.7%, SP: 85.7%); **CA-125:** AUC=0.692, 95%CI=0.525-0.858, P=0.034. Cut-off: 147U/ml (SE: 76.2%, SP: 71.4%). AUC: area under curve; CI: confidence interval; PSM: propensity score matching; U/ng: international unit per nanograms; U/ml: international unit per milliliters; SE: sensitivity; SP: specificity.

**Supplementary Figure 2.** ROC curves for determining CA-125 and CA-125/CEA cut-off in differential diagnosis: **A)** Ovarian cancer *VS* colorectal cancer (pre-PSM) – **CA-125/CEA:** AUC=0.858, 95%CI=0.728-0.989, P<0.001. Cut-off: 12.3U/ng (SE: 83.7%, SP: 77.8%); **CA-125:** AUC=0.599, 95%CI=0.430-0.769, P=0.312. Cut-off: 98.5U/ml (SE: 67.9%, SP: 66.7%); **B)** Ovarian cancer *VS* colorectal cancer (after PSM) – **CA-125/CEA:** AUC=0.753, 95%CI=0.516-0.990, P=0.070. Cut-off: 13U/ng (SE: 77.8%, SP: 77.8%); **CA-125:** AUC=0.543, 95%CI=0.260-0.827, P=0.757. Cut-off: 108.9U/ml (SE: 66.7%, SP: 66.7%); **C)** Advanced ovarian cancer *VS* colorectal cancer (pre-PSM) – **CA-125/CEA:** AUC=0.904, 95%CI=0.801-1.000, P<0.001. Cut-off: 22U/ng (SE: 88.5%, SP: 88.9%); **CA-125:** AUC=0.715, 95%CI=0.546-0.883, P=0.032. Cut-off: 98.5U/ml (SE: 82.3%, SP: 66.7%); **D)** Advanced ovarian cancer *VS* colorectal cancer (after PSM) – **CA-125/CEA:** AUC=0.889, 95%CI=0.717-1.000, P=0.005. Cut-off: 14U/ng (SE: 100.0%, SP: 77.8%); **CA-125:** AUC=0.605, 95%CI=0.331-0.879, P=0.453. Cut-off: 126.7U/ml (SE: 66.7%, SP: 66.7%). AUC: area under curve; CI: confidence interval; PSM: propensity score matching; U/ng: international unit per nanograms; U/ml: international unit per milliliters; SE: sensitivity; SP: specificity.
